# Smoking and Health Profiles of Hypertensive Patients with COVID-19: An Exploratory Study of Key Physiological Markers

**DOI:** 10.3390/jcm13237245

**Published:** 2024-11-28

**Authors:** Laura Haidar, Mara Amalia Bălteanu, Marius Georgescu, George Andrei Drăghici, Eveline-Anda Laza, Alina-Florina Șerb, Ramona Cioboată

**Affiliations:** 1 Department of of Functional Sciences, Physiology Discipline, Faculty of Medicine, “Victor Babeș” University of Medicine and Pharmacy, Eftimie Murgu Square No. 2, 300041 Timişoara, Romania; haidar.laura@umft.ro (L.H.); georgescu.marius@umft.ro (M.G.); 2 Center of Immuno-Physiology and Biotechnologies (CIFBIOTEH), Victor Babeș” University of Medicine and Pharmacy, Eftimie Murgu Square No. 2, 300041 Timişoara, Romania; 3 Department of Pulmonology, Faculty of Medicine, Titu Maiorescu University, 031593 Bucharest, Romania; mara.balteanu@prof.utm.ro; 4 Department of Toxicology, Faculty of Pharmacy, “Victor Babeș” University of Medicine and Pharmacy Timișoara, Eftimie Murgu Square No. 2, 300041 Timișoara, Romania; 5 Research Center for Pharmaco-Toxicological Evaluations, Faculty of Pharmacy, “Victor Babeș” University of Medicine and Pharmacy Timișoara, Eftimie Murgu Square No. 2, 300041 Timișoara, Romania; 6 The National Institute of Research—Development for Machines and Installations Designed for Agriculture and Food Industry (INMA), Bulevardul Ion Ionescu de la Brad 6, 077190 București, Romania; eveline_anda@yahoo.com; 7 Department of Biochemistry and Pharmacology, Biochemistry Discipline, “Victor Babeș” University of Medicine and Pharmacy Timișoara, Eftimie Murgu Square No. 2, 300041 Timișoara, Romania; aserb@umft.ro; 8 Pneumology Department, University of Medicine and Pharmacy, 200349 Craiova, Romania; ramona_cioboata@yahoo.com

**Keywords:** hypertension, smoking, COVID-19, clinical profile, cardiovascular markers, renal function, metabolic markers, inflammatory response

## Abstract

**Background/Objectives**: Smoking and hypertension are major contributors to cardiovascular diseases, with smoker hypertensives typically presenting with exacerbated health risks. These factors are associated with COVID-19 aggravation, but their cumulative impact in the context of this disease remains understudied. Our hypothesis was that hypertensive smokers display a more vulnerable health profile (versus non-smokers) upon hospital admission for COVID-19. **Methods**: This exploratory observational study compared the clinical profiles of hypertensive COVID-19 patients depending on their smoking status. Focusing on key cardiometabolic, blood, renal, hepatic, and inflammatory markers, this investigation included 100 hypertensive COVID-19 patients (50 smokers and 50 non-smokers) aged 50 and above. Logistic regression and Spearman’s correlations were used to identify significant predictors and relationships among variables. **Results**: Hypertensive smokers with COVID-19 were significantly more likely to exhibit higher heart rate (*p* = 0.047), left atrial size (*p* = 0.013) and diameter (*p* = 0.040), left ventricular end-systolic volume (*p* = 0.036), and interventricular septal thickness (*p* ≤ 0.001). These patients were also much more prone to display elevated CRP (*p* = 0.035) and hemoglobin (*p* = 0.011). The renal profiles of the smokers and non-smokers differed, with the smokers showing a significantly greater likelihood to have high serum urea (*p* = 0.036), but normal-to-low serum potassium (*p* = 0.011) and sodium (*p* ≤ 0.001). Their lipid profile was less favorable, with higher triglycerides (*p* ≤ 0.001), but lower HDL (*p* = 0.008). The strongest predictors of smoking status were interventricular septal thickness, triglycerides, and serum sodium. **Conclusions**: Hypertensive smokers admitted to the hospital with COVID-19 tend to exhibit a more adverse clinical profile, particularly in terms of cardiovascular remodeling, lipid imbalances, renal profile, and inflammation. These findings suggest that smoking exacerbates the effects of hypertension in the context of COVID-19, highlighting the need for more aggressive monitoring and management in this patient group.

## 1. Introduction

Smoking is a major yet modifiable risk factor for developing cardiovascular diseases [1,2]. This deleterious behavior promotes arterial stiffness, plaque formation, and chronic inflammation of the vascular endothelium, exerting cumulative, long-term effects on the circulatory system [3,4]. It also impacts both adaptive and innate immune responses, especially in older adults [5,6]. Such outcomes are particularly detrimental in the context of COVID-19 as smokers are more likely to experience severe respiratory symptoms, higher hospital admission rates, increased mortality, and post-acute complications [7,8]. On the other hand, a systematic review and meta-analysis analyzing data from 18 studies indicates that current smoking could be associated with lower hospitalization rates among individuals with COVID-19 [7].

A common chronic cardiac disorder, essential hypertension (also known as primary hypertension or idiopathic hypertension) is the most common form of hypertension. Characterized by persistently elevated blood pressure without a clearly identifiable underlying cause, this cardiovascular condition accounts for 90–95% of all adult hypertension cases [9]. With a rapidly increasing prevalence from the age of 50 years onward, it affects over half of the individuals aged 60–69 and about three-quarters of those aged 70 and older. Although smoking hypertensive patients typically exhibit worse health profiles than their non-smoking counterparts [4,10,11,12,13], very few studies have specifically focused on how these differences manifest in the context of COVID-19. However, these investigations have approached this topic from an epidemiological perspective, that is, focusing on the associations between smoking, hypertension, and the risk of COVID-19 and severity outcomes, and not on the health metrics separating these strata [14,15,16]. In this context, it is important to compare the clinical profile of hypertensive smokers and non-smokers having COVID-19 across various physiological markers.

Once infected with SARS-CoV-2, individuals are affected at multiple levels, with a wide variety of echocardiographic, hematologic, inflammatory, cardiometabolic, renal, and hepatic markers becoming altered [17,18]. Thus, left ventricular diastolic function and right ventricular function are perturbed following COVID-19 infection despite a normal left ventricular systolic function [19]. Hematological indices, including those related to Red Blood Cell Distribution Width (RDW), are also sensitive to SARS-CoV-2 insults [17,18]. Inflammatory markers, among others C-Reactive Protein (CRP) and erythrocyte sedimentation rate (ESR), can predict disease progression and clinical outcomes, being critical in COVID-19 management [17]. There is also evidence that changes in lipid profiles and glycemic disturbances are associated with worse outcomes in these patients [20,21]. Frequently observed during this illness, impaired kidney function serves not only as a marker, but also as a contributor to the severity of COVID-19 [22]. Furthermore, liver enzyme levels can be affected by this viral infection [23]. Understanding whether and how the health profile differs between hypertensive smokers and non-smokers in this context could provide valuable insights into the pathophysiological background of COVID-19 and have prognostic implications. This is especially important given the compounding effects of smoking and hypertension: both exacerbate COVID-19-related complications—including high inflammation, endothelial dysfunction, and altered immune regulation [14,16,17].

Based on the aforementioned data, our hypothesis was that hypertensive smokers display a more vulnerable health profile (versus non-smokers) upon hospital admission for COVID-19. Studying this group is crucial since it may face a uniquely high risk of severe illness and complications. By focusing on clinical profiles at admission, we aimed to consistently control for baseline factors and offer a clear comparative analysis. We compared key cardiac, blood, kidney, metabolic, and inflammation markers to identify the most critical factors that differentiate these groups. Determining the specific features of these cohorts may provide relevant data for better separation of the clinical phenotypes of COVID-19 in hypertensive patients [7,14]. This knowledge should also help to understand how smoking amplifies the adverse effects of hypertension in the setting of COVID-19, potentially leading to better risk stratification and improved patient management.

## 2. Materials and Methods

### 2.1. Design

We conducted a single-site, observational, exploratory clinical study without industry support to collect preliminary data on the physiological markers with the potential to delineate between non-smoking and smoking hypertensives with COVID-19. This investigation was conducted at the “Victor Babeş” Clinical Hospital of Infectious Diseases and Pneumology, located in Craiova (Dolj county, Romania) [24], in agreement with the Declaration of Helsinki (1964) and the subsequent amendments. The study was approved by the Institutional Ethics Committee (IEC) at this medical facility (approval No. 13111/18.09.2024). Data for the patients included in this study were collected between September, 2024 and 15 October 2024. Available data support that the prevailing SARS-CoV-2 variant circulating during this time period was KP.3. of the Omicron family [25]. Informed consent was obtained from all the patients or their caregivers, with all the identifying information being kept confidential.

### 2.2. Protocol

The study population involved hypertensive patients aged 50 years or older admitted to hospital due to COVID-19. Two practical arguments lay behind our choice to select this age range for enrollment. The pool of eligible participants in hospitals is larger among older patients given the age-related increase in the prevalence of primary hypertension and smoking [4,26,27]. In addition, smoking-related damage is likely more substantial in older individuals due to longer tobacco exposure, making them a particularly high-risk group of cardiac patients [1,28].

In this study, we focused on patients specifically at the time of hospital admission. All the analyses were, hence, performed as close as possible to this initial point to ensure that our data sets accurately reflect the patients’ baseline status upon entry. This approach aimed at capturing their initial physiological and clinical profiles without considering the severity of their COVID-19 symptoms at later stages. This framework also minimized variability introduced by differing treatment responses, the progression of symptoms, and in-hospital interventions, thereby allowing us to analyze baseline characteristics consistently across all the participants. Moreover, it enabled a clearer assessment of the potential influence of smoking and hypertension on the clinical markers observed at the initial point of care.

The main inclusion criteria were age 50 years and above, COVID-19 infection confirmed via a positive PCR test or rapid antigen test, hospitalization lasting at least until completion of all study-related analyses, a known history of primary hypertension, stable medication use for at least three months prior to enrollment, signed informed consent, and a documented smoking status. Non-smokers (never smokers) refer to adults who have never smoked cigarettes (or other tobacco products) or smoked less than 100 cigarettes in their lifetime [29]. Current smokers include adults who have smoked 100 cigarettes in their lifetime and who currently smoke cigarettes (or other tobacco products) [29]. Ex-smokers (former smokers), defined as adults who have smoked more than 100 cigarettes (or other tobacco products) during their lifetime but had quit till enrollment [29], were excluded from the study. This aimed to minimize confounding factors related to smoking cessation, decrease variability, and improve the clarity of the results obtained. We also did not consider the severity of COVID-19 because the present investigation aimed to maintain a focused analysis of smoking’s influence on hypertensive patients with COVID-19, without the confounding complexities that would arise from including severity as a factor (e.g., different treatment approaches, time–disease progression, and varying inflammatory or immune responses).

Other major exclusion criteria were secondary hypertension (e.g., renal artery stenosis, hyperaldosteronism, and pheochromocytoma); a history of myocardial infarction, stroke, heart failure, or any major cardiovascular event within the past six months; having undergone major cardiovascular procedure (e.g., coronary artery bypass graft and stent placement) within the last 60 days; chronic kidney disease (CKD) stages 3–5 (eGFR <60 mL/min/1.73 m^2^) or requiring dialysis; severe infections that could affect inflammatory markers; active malignancy or a history of cancer treatment within the past 5 years; advanced liver diseases such as cirrhosis or acute hepatic failure; the use of immunosuppressive therapy or corticosteroids; severe psychiatric disorders (e.g., schizophrenia and bipolar disorder) or cognitive impairment (e.g., dementia); and a history of drug or alcohol abuse that could interfere with adherence to study protocols.

### 2.3. Measurements

First, 175 hypertensive patients aged 50 years and above were pre-enrolled on a “first come, first served” basis after being admitted to the hospital with a COVID-19 diagnosis. Our study was conducted on patients with COVID-19, irrespective of severity. The analyses were performed as soon as possible after admission. The patients remained in the hospital after COVID-19 confirmation at least until all the analyses were completed. Their subsequent status (whether they were discharged or remained hospitalized), as well as the treatment received and their outcomes, were not considered in this study.

Systolic blood pressure (SBP), diastolic blood pressure (DBP), and heart rate (HR) were determined at admission, whereas blood samples (50 mL) were obtained as soon as possible, typically 1–2 h post-admission. Echocardiography was performed within 24–48 h following hospital admission to evaluate the patient’s cardiac function. Ejection fraction (EF), left atrial size (LAS), left atrial diameter (LAD), left ventricle diameter (LVD), left ventricular end-diastolic volume (LVEDV), left ventricular end-systolic volume (LVESV), interventricular septal thickness (IVSd), and pulmonary artery systolic pressure (PASP) were the echocardiographic indicators taken into account in this study. These metrics are indicative of whether and how smoking might potentiate the cardiovascular effects of hypertension in COVID-19 patients aged 50 years and above. Briefly, changes in HR combined with high blood pressure are helpful for evaluating the overall cardiovascular strain. EF relates to cardiac performance, whereas elevated LAS and LAD can suggest heart problems, including atrial fibrillation and stroke risk. LVD and LVEDV are markers of ventricular hypertrophy or dilation and increased preload, respectively. An elevated LVESV points to worsening heart failure, whereas increased IVSd is typically associated with hypertrophic changes due to hypertension. Heightened PASP is often related to heart failure, valvular disease, or lung-related issues [30].

Erythrocyte sedimentation rate (ESR), C-Reactive Protein (CRP), hemoglobin, and platelet-related indices (RDW-CV and RDW-SD) were the hematological markers analyzed. Renal function was evaluated as a function of serum creatinine, serum urea, serum uric acid, serum sodium, and serum potassium. The cardiometabolic markers measured were related to lipid profile (i.e., total cholesterol, LDL, HDL, and triglycerides) and glycemic function (i.e., random glucose and glycated hemoglobin—HbA1c). Liver function was assessed via aspartate aminotransferase (AST) and alanine aminotransferase (ALT). Sociodemographic data (sex, age, origin, and smoking status), as well as data on the presence of diabetes and obesity, were also collected [31]. An HbA1c above 6.5% and/or fasting plasma glucose greater than 125 mg/dL and/or random plasma sugar exceeding 200 mg/dL were used for diagnosing new diabetes [32]. Obesity was defined as a body mass index (BMI) of 30 kg/m^2^ or higher [33]. To ensure a homogeneous study group and reduce bias, 100 patients were recruited to the study after careful matching for age, sex, and smoking status, and then stratified based on their smoking status (as defined above). This practical and effective approach allowed us to maximize the precision while reducing noise and maintaining sufficient statistical power [34].

### 2.4. Data Analysis

Statistical analysis was conducted using the Statistica version 8 software (StatSoft Inc., Tulsa, OK, USA). Fisher’s exact tests were employed to assess inter-strata differences for categorical variables. A Mann–Whitney U-test was used to validate inter-group homogeneity with respect to age [35]. Data for continuous variables are given as median values with lower and upper quartiles, not as means with one standard deviation. This approach is frequently used in exploratory studies because it provides a more robust, interpretable, and distribution-flexible summary of data, which is particularly valuable when patterns and distributions are not yet fully understood [34].

Logistic regression—a method recommended for dichotomous variables—was next applied to continuous outcomes to identify the most important separators delineating smoker from non-smoker hypertensives with COVID-19 [36]. Both the Wald test and the Type 3 Likelihood Ratio (LR) test are commonly employed in logistic regression models [37]. We chose the latter approach since this technique evaluates the contribution of each variable while controlling for the effect of all the other variables in the model. This is very important in the case of multicollinearity, i.e., multiple predictors (e.g., age, cholesterol, heart rate, and other health indicators) that may interact or correlate with each other [35]. The direction and the magnitude of the association between significant predictor variables and the smoking status were determined based on the values of parameter estimate (direction) and chi-square (magnitude) [37,38]. A chi-square value of 6.64 was set as a cutoff to identify the most influential predictor variables. These values correspond to a *p* value less than 0.01—a significance threshold that is often used for determining stronger predictors [37]. Finally, intra-strata Spearman’s correlations were conducted for these variables and the other parameters analyzed. This aimed to identify association patterns that may be unique to smokers or non-smokers. The strength of these associations was described as follows: 0.00 ≤ ∣*r_s_*∣ <0.30: weak correlation; 0.30 ≤ ∣*r_s_*∣ < 0.70: moderate correlation; and 0.70 ≤ ∣*r_s_*∣ < 1.00: strong correlation [34]. A two-sided *p* value less than 0.05 was considered significant [38].

## 3. Results

### 3.1. Health Profiles of Hypertensive Non-Smokers and Smokers with COVID-19

After age- and sex-matching, we enrolled 50 hypertensive smokers and 50 hypertensive non-smokers with COVID-19. Their sociodemographic characteristics are presented in Table 1. No significant inter-strata differences existed for sex distribution, area of origin, diabetes, or obesity status (Table 1). Median values for the continuous parameters (including the lower and upper quartiles) are given in Table 2. The non-smoking and smoking hypertensive patients with COVID-19 were of similar age (Mann–Whitney tests, *p* = 0.292). The median values of cardiac parameters were typically within or close to the normal range, tending to be higher in the smokers than in the non-smokers (Table 2).

The median values for CRP and HbA1c were above the normal range for both strata, as well as for ESR in the smoking patients (Table 2). An altered renal profile (versus normal physiological values) was identified for serum urea and especially for serum uric acid, with the smokers displaying higher values than the non-smoker hypertensive patients with COVID-19 (Table 2). The other renal and glycemic parameters analyzed, as well as the blood, metabolic, and liver health metrics, were within the normal limits (Table 2).

The key parameters of the Type 3 (LR) test are given in Table 3. Age was a significant predictor of smoking status, with older individuals being more likely to be non-smokers (Table 3). Although the systolic blood pressure, diastolic blood pressure, and ejection fraction were not significantly associated with smoking status, elevated heart rate was associated with lower odds of being a non-smoker (Table 3). Patients with larger left atrial size and left atrial diameter were also less likely to be non-smokers (Table 3). Similar associations were found for the left ventricular end-systolic volume and interventricular septal thickness, but not for the other echocardiographic metrics. Platelet-related parameters and the erythrocyte sedimentation rate were not significant predictors of smoking status (Table 3). Higher CRP and elevated hemoglobin levels, by contrast, were also associated with lower odds of being a non-smoker (Table 3). However, glycemic parameters did not account for differences between the smokers and non-smokers (Table 3).

Serum urea, serum sodium, and serum potassium were significant separators between the non-smoking and smoking patients having primary hypertension and COVID-19 (Table 3). Higher serum urea was associated with lower odds of being a non-smoker, but this variable was less predictive compared to serum sodium and serum potassium (Table 3). For the latter outcomes, lower levels are associated with a higher probability of being a smoker (Table 3). In contrast, no significant associations were found for the other renal markers, as well as for hepatic parameters and LDL (Table 3). However, higher HDL and total cholesterol were associated with higher odds of being a non-smoker (Table 3). On the other hand, elevated triglycerides were associated with a lower likelihood of not smoking (Table 3).

The strongest predictors of smoking status were, by far, IVSd, serum sodium, and triglycerides, all showing chi-square values well above 6.64 (Table 3). Age, LAS, hemoglobin, serum potassium, total cholesterol, and HDL played a moderate role in separating smokers from non-smokers, with the latter two variables being above the cutoff value for influential variables. On the other hand, HR, LVESV, LAD, CRP, and serum urea had only a minor importance in predicting smoking status despite reaching statistical significance.

### 3.2. Correlational Patterns of Non-Smokers and Smokers

Based on the aforementioned data, inter-strata correlational patterns were investigated for IVSd, serum sodium, and triglycerides—the most influential variables to distinguish the smokers from the non-smokers. The corresponding Spearman’s correlations are shown in Table 4. Most significant associations were of moderate strength (Table 4). The strongest correlation was observed between IVSd and LVEDV in both the smokers and non-smokers, which is a moderate-to-high positive association (Table 4). The former variable also correlated directly with serum sodium in both strata (Table 4). Positive associations were identified between IVSd and age, serum urea, serum creatinine, and serum sodium, but only for the smokers (Table 4).

In both strata, serum triglycerides correlated directly with ejection fraction, hemoglobin levels, and total cholesterol, but negatively with left atrial diameter (Table 4). This lipid metric showed an inverse relationship with PSAP in the non-smoking hypertensives with COVID-19 (Table 4). In contrast, triglycerides displayed in smokers had negative associations with left ventricle diameter, RDW-SD, and ALT (Table 4). Serum sodium showed a positive association with ejection fraction and a negative association with left ventricle diameter, but only in the smoking patients (Table 4). The other associations did not reach the threshold for statistical significance (Table 4).

## 4. Discussions

Although the individual effects of smoking and hypertension on COVID-19 risk, progression, and severity are well documented [7,8,14,15,16,18,19,20], little information exists regarding how these factors interact in the same patient. The clinical profile of hypertensive individuals admitted to hospitals with this illness is also poorly understood [14,15]. Moreover, the markers distinguishing smokers from non-smokers in this high-risk group or the potential mechanisms underlying these differences remain insufficiently explored [15]. This study addresses these gaps by combining logistic regression with Spearman’s correlations, thus leveraging the strengths of both methods—identifying significant predictors and then exploring how they relate to other key variables [34,36,37].

Medical evidence provides robust evidence for the existence of a strong connection between hypertension and both obesity and diabetes [15,16,26]. In our study, no inter-strata differences existed in the incidence of diabetes or obesity. One can, hence, expect that the presence of these conditions likely did not affect our results. We also found a trend for older individuals to be less likely to smoke. Epidemiological evidence shows that in recent decades, more people have become aware of smoking hazards and adopted health-conscious habits, especially as they age [3,39]. This may account for the aforementioned findings. Alternatively, it is plausible that this trend reflects survivor bias, with smokers exhibiting higher mortality rates within this age bracket [40].

Hypertensive smokers with COVID-19 were more prone to showing an adverse cardiac profile, with elevated heart rate, left atrial size and diameter, left ventricular end-systolic volume, and interventricular septal thickness. Indeed, the stimulatory effect of smoking on the sympathetic nervous system and the vasoconstrictor effect of nicotine and other chemicals in cigarettes are known to raise heart rates in smokers [41,42]. However, this physiological response could indicate an additive effect since tachycardia is common in COVID-19 due to systemic inflammation, fever, and hypoxia [43]. Such outcomes are a major problem for high-risk groups, including older individuals with pre-existing cardiovascular conditions. One may, hence, expect that the cumulative effect of smoking, hypertension, and COVID-19 will exacerbate cardiovascular strain in this vulnerable population, leading to poorer outcomes and long-term complications [14].

Smoking is a potent trigger of cardiac remodeling even in healthy individuals without other cardiovascular risk factors. It was found that smokers display lower atrial reservoir and conduit strains, as well as worse ventricular functions, when compared to non-smoking individuals [44,45,46]. These changes predispose them to adverse cardiac conditions such as atrial fibrillation, atrial cardiomyopathy, and diastolic heart failure [1]. On the other hand, some of these modifications in heart morphometry may be associated with COVID-19 infection. Thus, both left and right ventricular dysfunction—as assessed via ejection fraction or strain measurements—were reported in COVID-19 patients with myocardial injury, although they were temporary, subsiding at two months post-infection [47].

Interventricular septal thickness emerged as the most significant cardiac variable and one of the strongest predictors of smoking status among the analyzed markers. Clinical evidence supports that septal thickening is not the simple reactive outcome of long-term exposure to high blood pressure, but most likely actively contributes to its development [48,49]. From this point of view, the positive correlations observed here between the left ventricular end-diastolic volume and interventricular septal thickness in both strata may reflect a compensatory mechanism by which the heart adapts to the increased workload by thickening the septum and expanding the left ventricle [50,51,52]. Indeed, mechanical models support that the septum plays a role in transferring energy between the ventricles, affecting both preload and afterload conditions, which in turn can influence LVEDV [53].

Our results support that hypertensive smokers with COVID-19 are likely to display a thicker interventricular septum wall than their non-smoking counterparts. In line with evidence-based medicine, septal thickening exhibits the strongest association with smoking status and history among the evaluated cardiac markers [54]. This remodeling process is typically a progressive process that takes years to develop [51,52]. In contrast, COVID-19 primarily affects cardiac function acutely without causing structural changes such as septal hypertrophy [55]. However, it can occur more rapidly in populations with pre-existing cardiovascular disease, especially when triggered by acute events like a heart attack or severe infection [56]. Of note, correlational analysis provides evidence for a connection between the thickening of the interventricular septum and the severity of renal dysfunction in smokers with COVID-19—as supported by direct associations between the former variable and the serum urea, creatinine, and sodium levels. This aligns with the literature data on the interplay between smoking, hypertension, and kidney function [56,57].

It was reported that smokers tend to display higher CRP and hemoglobin than non-smokers [4,58,59]. This is consistent with our results. Thus, elevated CRP and hemoglobin were associated with a higher probability of being a smoker, despite showing ESR and platelet metrics similar to non-smoking individuals. CRP is generally considered a more sensitive marker for acute inflammatory response, like that caused by bacterial or viral insults, than for chronic inflammation, such as smoking-related inflammation [58]. This increase in CRP is, therefore, most probably connected to COVID-19 infection. On the other hand, hemoglobin rise is a common condition in smoking, being linked to its well-known hypoxic effect [59]. This response may be amplified in smokers with COVID-19, as the infection further impairs oxygen exchange, pushing the body to increase red blood cell production [43,60]. Importantly, clinical evidence points to the compounded effect of increased CRP and hemoglobin levels as a prognostic factor for worse outcomes in the context of this disease [16,17,61].

Studies have indicated that smokers typically have a more deranged renal profile, with reduced blood flow, increased risk of chronic kidney disease, elevated proteinuria, lower GFR, and heightened oxidative stress [4,10,62,63]. Hypertensive smokers with COVID-19 showed a distinct renal profile, exhibiting a tendency towards higher serum urea, but lower serum sodium and serum potassium. Data on the former parameter align with medical research outcomes [62]. Although less clear, the renal regulation of sodium and potassium appears to be independent of smoking status [63]. On the other hand, essential hypertension is often associated with an imbalance in electrolyte levels, particularly persistently high sodium and low potassium levels [64]. Taken together, these results indicate that the differences observed here in electrolyte balance between hypertensive smokers and non-smokers could be at least partly due to COVID-19 infection. Similar data have not yet been reported although renal function impairment is often encountered in these patients [22,65].

Serum sodium was the most influential variable separating non-smokers from smokers. The pattern of decreased, but low-normal serum sodium levels in the latter strata can be associated with inflammatory response, renal imbalance, and stress [66]. Such responses have been attested in COVID-19, with mildly hyponatremic adults having a worse prognosis than those without electrolyte imbalance, including high normal values [67]. For example, Guan et al. (2020) reported a mean serum sodium of 138 mmol/L (range 135–141 mmol/L) in 1099 patients hospitalized for COVID-19 in China [68]. A study with 5700 hospitalized patients with COVID-19 in New York found similar values, which is an average sodium level of 136 mmol/L (133–138 mmol/L) [69]. These values are consistent with those observed in this study for hypertensive smokers. It is also worth mentioning that even mild hyponatremia (low–normal values) is a predictor of worse outcomes in patients with cardiovascular diseases [70]. As a result, smoking adults with essential hypertension may be at a greater risk of complications or severe COVID-19 compared to their non-smoking counterparts.

The positive correlation between serum sodium and interventricular septum thickness in both strata is most probably related to sodium-induced cardiac hypertrophy—a common condition in essential hypertension [9]. The similar association between serum sodium and ejection fraction in smokers may reflect the heart’s effort to compensate more aggressively in response to increased blood viscosity and sodium-induced volume overload [1,4,9]. The inverse relationship between serum sodium and left ventricle diameter in the same strata, on the other hand, could indicate a more accelerated progression towards left ventricle enlargement due to the effects of high blood pressure and the vasoconstrictive properties of smoking [71].

Evidence from research connects smoking to lipid dyshomeostasis, emphasizing its impact on disrupting lipid balance [1,4,72,73,74,75]. In this study, the smoking hypertensive patients with COVID-19 were prone to exhibiting a more adverse lipid profile, with lower HDL, but higher triglycerides. This is consistent with the literature data [72,73]. The most influential parameter in separating the two strata was triglyceride levels—a hallmark of insulin resistance, metabolic syndrome, and elevated cardiovascular risk [74]. This lipid imbalance is also linked to heightened mortality in COVID-19 patients, being associated with greater inflammation and compromised immune function—both of which exacerbate the severity of COVID-19 [75].

While differences in triglycerides and HDL between the groups can be attributed to smoking status, the higher total cholesterol in the non-smokers seems counterintuitive. However, elevated total cholesterol may hint at enhanced immune cell function as a protective mechanism or adaptation during illness or inflammation, rather than at a simple lipid metabolism difference. In fact, a systematic review and meta-analysis found that lower total, HDL, and LDL cholesterol are strongly linked to COVID-19 severity and mortality [76].

Considering the available evidence, it is difficult to determine the clinical relevance of the observed correlations for triglycerides in both strata, i.e., direct associations with ejection fraction, hemoglobin levels, and total cholesterol, and an inverse relationship with left atrial diameter. Being independent of smoking status, these associations may be a simple extension of the altered cardiometabolic profile of hypertensive patients [9,76]. For example, higher triglycerides and total cholesterol are associated with a worse cardiovascular profile [77]. In addition, triglycerides are associated with heart failure and myocardial injury in COVID-19 patients, with elevated concentrations correlating with unfavorable cardiac outcomes [76]. In contrast, relevant data on the other interactions are not yet available.

Stratum-specific correlations for triglycerides differed between the smokers and non-smokers. The negative associations of triglycerides with left ventricle diameter, RDW-SD, and ALT in the smokers hint at the dysregulation of lipid metabolism in these patients—possibly associated with smoking-related damage to the heart, blood, and liver, and mediated via insulin resistance [4,10,78]. This is a cause of concern since COVID-19 could amplify the existing cardiovascular, hematologic, and hepatic dysregulation related to lipid metabolism in smokers [19,20,21], leading to worse outcomes.

Overall, our results indicate that smoking and non-smoking hypertensive patients with COVID-19 reveal distinct clinical phenotypes, particularly with respect to cardiometabolic and renal profiles. Smokers tend to exhibit a more unfavorable cardiac risk profile, with increased heart rate, left atrial size and diameter, left ventricular end-systolic volume, and interventricular septal thickness. These individuals are also more prone to display impaired lipid metabolism, exhibiting lower HDL and higher triglycerides. Furthermore, smoking is associated with a higher likelihood of having elevated serum urea, but low-normal serum sodium and potassium. Among the investigated markers, interventricular septal thickness, serum sodium, and triglycerides were the strongest predictors of smoking status in hypertensive patients aged 50 years or above upon hospital admission for COVID-19.

Several limitations of this investigation need to be discussed. First, we conducted a cross-sectional study focusing on the clinical profiles of hypertensive COVID-19 patients depending on their smoking status at the time of hospital admission. As a result, causal relationships between smoking, hypertension, and COVID-19 outcomes cannot be established, nor can we dismiss the possibility of potential reversed causation. For example, it is unclear if altered lipid metabolism is a cause or an effect of these conditions. This study, however, determines significant associations between key health markers and smoking status in hypertensive COVID-19 patients, thus providing a solid foundation for future longitudinal research to explore causality more thoroughly.

Second, the findings of this study might not reflect broader population trends since it was conducted at one hospital in Romania and included 100 patients. Nonetheless, this single-site approach with a moderate sample size is commonly used in pilot exploratory studies, as was the case of our study, since it allows for controlled variables and consistent data collection [79]. In addition, age- and sex-matching ensures excellent inter-strata comparability, with the results providing a focused analysis of the groups [79]. Moreover, the sample size used (50 individuals per group) allowed us to conduct a controlled comparison between strata, supporting the primary goal of the present study: to identify significant markers and trends rather than to make generalizable conclusions or detect small effect sizes [80].

Third, we did not include former smokers. However, this approach allowed us to avoid confounding factors from smoking cessation effects, leading to an analysis more focused on active smokers. This decision creates a clearer distinction between active smokers and non-smokers, avoiding complexities that could arise from the varying duration and health effects of smoking cessation.

Fourth, this study did not consider the severity of COVID-19 or its treatment. However, we focused on the clinical profile of the patients at the time of hospital admission rather than focusing on treatment and outcomes. This framework enabled us to capture the initial features that might predispose patients to different disease trajectories without the variability introduced by treatment protocols (dependent on disease progression, hospital resources, and physician discretion). By excluding these factors, our research avoids the complexities of different treatment regimens and inflammatory responses that might have confounded the results. Moreover, the inclusion of COVID-19 severity and treatment would require subgrouping and multivariable adjustments, thus adding considerable complexity—a thing to avoid in pilot studies.

Finally, our work lacks long-term follow-up. We, hence, cannot determine the effect of smoking on long-term recovery or post-acute complications in hypertensive patients admitted to the hospital with COVID-19. However, this study is exploratory, being focused on identifying immediate physiological differences between smokers and non-smokers at hospital admission. Future studies should use these findings as a basis for more comprehensive research that includes long-term tracking. This current study is intended as an initial profile comparison, and we agree that a future study incorporating treatment and outcomes could further clarify these relationships. Moreover, focusing on short-term outcomes allows the study to provide actionable data in the acute care setting, which is highly relevant during a pandemic.

## 5. Conclusions

Focusing on key cardiometabolic, renal, and inflammatory markers, this pilot exploratory study has examined the clinical profiles of hypertensive COVID-19 smokers and non-smokers aged 50 years and above. Smokers were more likely to display higher heart rates, larger left atrial size/diameter and left ventricular end-systolic volume, and increased interventricular septal thickness—suggestive of greater cardiovascular strain. The same patients were prone to display elevated levels of triglycerides and lower HDL levels. Smokers also tended to have higher serum urea and lower sodium levels, pointing to potential kidney dysfunction. CRP levels and hemoglobin levels showed similar trends. Interventricular septal thickness, serum sodium, and triglycerides were the strongest predictors of smoking status among the health metrics analyzed. These findings suggest that hypertensive smokers with COVID-19 present with a worse clinical profile compared to their non-smoking counterparts, particularly with respect to cardiac, lipid, and renal markers.

## Figures and Tables

**Table 1:** Sociodemographic characteristics in hypertensive non-smokers and smokers with COVID-19.

Characteristic	Strata	Non-Smokers	Smokers	*p*
Sex	Male	31 (62%)	38 (76%)	0.131
	Female	19 (38%)	12 (24%)	
Origin	Rural	23 (46%)	21 (42%)	0.840
	Urban	27 (54%)	29 (58%)	
Diabetes	Yes	19 (38%)	21 (42%)	0.838
	No	31 (62%)	29 (58%)	
Obesity	Yes	8 (16%)	6 (12%)	0.774
	No	42 (84%)	44 (88%)	

Data are shown as absolute values with the corresponding percentages in parentheses.

**Table 2:** Measured values for selected parameters in hypertensive non-smokers and smokers with COVID-19.

Characteristic	Non-Smokers	Smokers	Reference Range
Age (years)	71 (67; 78)	69 (62; 78.5)	
SBP (mm Hg)	140 (130; 154)	147 (130; 158)	90–130
DBP (mm Hg)	80 (80; 90)	80 (75; 90)	60–80
HR (bpm)	85 (77; 95)	97 (81; 117)	60–100
EF (%)	47 (33; 57)	40 (25; 50)	50–70
LAS (mm)	42 (37.25; 45)	44.5 (40; 49)	<41
LAD (mm)	42 (37; 46)	44 (41; 81)	25–53
LVD (mm)	46 (44; 57)	50 (42; 57)	39–59
LVEDV (mL)	110 (102.5; 121)	115 (105; 125)	46–150
LVESV (mL)	70.5 (48.5; 1089.5)	72.5 (53.25; 97.75)	14–61
IVSd (cm)	1.1 (1; 1.2)	1.2 (1; 1.3)	0.6–1.2
PSAP (mm Hg)	48.5 (38.25; 60.75)	50 (38; 65)	<40
RDW-CV (%)	14.75 (14.1; 15.87)	15.25 (14.15; 15.87)	11.5–15.4
RDW-SD (fL)	45.8 (44.12; 49)	47.35 (41.15; 47.95)	39–46
ESR (mm/h)	26 (14; 43.5)	32.5 (20; 47)	0–30
CRP (mg/L)	13 (9; 22)	12 (6; 21)	<10
Hemoglobin (g/dL)	12.2 (11.2; 13.7)	13.5 (11.7; 14.9)	12.1–17.2
Random glucose (mg/dL)	130 (113; 161)	134 (110; 166)	<200
HbA1c (%)	6.7 (6; 7.8)	6.85 (6; 7.95)	<6.5
Serum urea (mg/dL)	46.5 (38.25; 64.2)	52.5 (41.75; 68)	<49
Serum uric acid (mg/dL)	7.71 (6.2; 10.3)	8.4 (7; 10.5)	3–7
Serum creatinine (mg/dL)	0.98 (0.87; 1.25)	1.06 (0.92; 1.35)	0.6–1.3
Serum sodium (mmol/L)	146 (132.25; 147)	138 (136; 140)	135–147
Serum potassium (mmol/L)	4.3 (4; 4.6)	4.2 (4; 4.6)	3.6–5.2
Total cholesterol (mg/dL)	162 (116; 182)	150 (116; 285)	<200
LDL (mg/dL)	94 (73; 123)	93 (73; 124)	<130
HDL (mg/dL)	43 (36; 49)	39 (33; 47)	>40
Triglycerides (mg/dL)	104 (88; 152)	135 (97; 180)	<150
AST (UI/L)	27.5 (18.25; 39.75)	29 (22.25; 41.75)	5–56
ALT (UI/L)	25 (16.25; 36.25)	25.5 (18.25; 36.75)	9–40

Data are presented as median values with lower and upper quartiles (in parentheses).

**Table 3:** Logistic regression results: predictors of non-smoking status.

Variable	Estimate	Chi-Square	*p*
Age	0.124	6.25	0.013 *
SBP	−0.01	1.17	0.616
DBP	0.012	0.25	0.710
HR	−0.029	3.94	0.047 *
EF	0.021	0.44	0.505
LAS	−0.164	6.16	0.013 *
LAD	−0.092	4.19	0.040 *
LVD	−0.064	1.45	0.228
LVEDV	0.004	0.26	0.608
LVESV	−0.023	4.37	0.036 *
IVSd	−10.465	11.41	<0.001 ***
PSAP	0.02	0.91	0.340
RDW-CV	−0.12	1.60	0.206
RDW-SD	−0.151	2.96	0.085
ESR	−0.015	0.42	0.518
CRP	−0.061	4.43	0.035 *
Hemoglobin	−0.486	6.63	0.011 *
Random glucose	0.004	0.52	0.472
HbA1c	0.075	0.06	0.819
Serum urea	−0.059	4.66	0.036 *
Serum uric acid	0.075	0.22	0.639
Serum creatinine	−0.234	0.03	0.869
Serum sodium	0.228	13.30	<0.001 ***
Serum potassium	1.594	6.43	0.011 *
Total cholesterol	0.045	9.47	0.002 **
LDL	−0.085	0.57	0.452
HDL	0.010	6.98	0.008 **
Triglycerides	−0.022	16.99	<0.001 ***
AST	−0.023	2.33	0.127
ALT	0.019	3.19	0.074

Marked values (*) show significant differences compared to non-smoker hypertensives with COVID-19 (Type 3 LR test, ***—*p* < 0.001, **—*p* < 0.01, and *—*p* < 0.05).

**Table 4:** Intra-strata correlational analysis.

	Non-Smoker			Smoker		
	IVSd	Serum Sodium	Triglycerides	IVSd	Serum Sodium	Triglycerides
Age	−0.03	−0.21	−0.05	0.34 *	0.06	−0.03
SBP	0.16	−0.10	−0.12	−0.06	0.05	0.07
DBP	0.04	0.13	−0.02	−0.04	0.19	0.26
HR	0.06	0.16	0.04	0.03	0.25	0.05
FE	−0.03	−0.13	0.32 *	0.16	0.31 *	0.44 *
LAS	0.05	−0.18	0.01	0.05	0.03	0.05
LAD	−0.10	−0.10	−0.28 *	−0.15	−0.11	−0.31 *
LVD	0.12	0.05	0.09	−0.08	−0.30 *	−0.31 *
LVEDV	0.68 *	0.02	0.08	0.63 *	0.16	0.21
LVESV	0.06	−0.28	0.07	0.11	0.11	−0.11
IVSd	1.00	0.33 *	0.19	1.00	0.32 *	0.03
PSAP	−0.03	−0.01	−0.32 *	0.16	−0.01	−0.25
ESR	0.23	0.12	0.17	0.13	0.21	−0.04
CRP	0.04	0.09	−0.24	0.10	−0.11	0.03
Hemoglobin	0.04	−0.02	0.29 *	−0.08	0.05	0.35 *
RDW-CV	−0.03	0.21	−0.20	0.10	−0.03	−0.42 *
RDW-SD	−0.01	0.27	−0.19	0.05	0.12	−0.26
Hemoglobin	0.11	0.10	0.02	0.02	0.04	0.18
Random glucose	0.08	0.01	−0.02	0.04	−0.10	0.15
Serum urea	0.00	0.20	−0.18	0.31 *	0.07	−0.35 *
Serum uric acid	−0.17	−0.04	−0.18	−0.05	−0.17	0.11
Serum creatinine	0.14	0.21	−0.22	0.36 *	0.11	−0.12
Serum sodium	0.33 *	1.00	0.15	0.32 *	1.00	0.16
Serum potassium	0.20	0.05	−0.18	0.04	−0.09	0.10
Total cholesterol	0.00	−0.06	0.36 *	0.22	0.12	0.35 *
HDL	−0.11	−0.21	−0.05	0.28	0.09	−0.03
LDL	0.10	−0.07	0.17	0.20	0.14	0.17
Triglycerides	0.19	0.15	1.00	0.03	0.16	1.00
AST	−0.06	0.11	−0.13	0.09	−0.04	−0.48 *
ALT	0.04	0.15	−0.08	0.02	0.01	−0.20

Bold and marked values (*) indicate significant associations (Spearman’s correlations, *—*p* < 0.05).

## Data Availability

All the data generated or analyzed during this study are included in this published article.
